# Advancements in Laser Wire-Feed Metal Additive Manufacturing: A Brief Review

**DOI:** 10.3390/ma16052030

**Published:** 2023-03-01

**Authors:** Mohammad Abuabiah, Natago Guilé Mbodj, Bahaa Shaqour, Luqman Herzallah, Adel Juaidi, Ramez Abdallah, Peter Plapper

**Affiliations:** 1 Mechanical and Mechatronics Engineering Department, Faculty of Engineering and Information Technology, An-Najah National University, Nablus P.O. Box 7, Palestine; 2 Department of Engineering, University of Luxembourg, 6, Rue-Kalergi, L-1359 Luxembourg, Luxembourg

**Keywords:** laser wire additive manufacturing, monitoring, control, modelling, parametric, path planning

## Abstract

Laser Wire-Feed Metal Additive Manufacturing (LWAM) is a process that utilizes a laser to heat and melt a metallic alloy wire, which is then precisely positioned on a substrate, or previous layer, to build a three-dimensional metal part. LWAM technology offers several advantages, such as high speed, cost effectiveness, precision control, and the ability to create complex geometries with near-net shape features and improved metallurgical properties. However, the technology is still in its early stages of development, and its integration into the industry is ongoing. To provide a comprehensive understanding of the LWAM technology, this review article emphasizes the importance of key aspects of LWAM, including parametric modeling, monitoring systems, control algorithms, and path-planning approaches. The study aims to identify potential gaps in the existing literature and highlight future research opportunities in the field of LWAM, with the goal of advancing its industrial application.

## 1. Introduction

Additive manufacturing (AM), also known as 3D printing or rapid prototyping, is a process of creating a physical object by adding layers of material on top of each other. In the past, AM was mainly used for creating visual representations of products as they were being developed. However, with advances in material science, it is now possible to create more durable and accurate pieces using AM without wasting material as is common in traditional subtractive manufacturing processes. AM is used in a variety of industries, including the automotive, aerospace, medical, dental, and fashion sectors [1].

**Figure 1:**
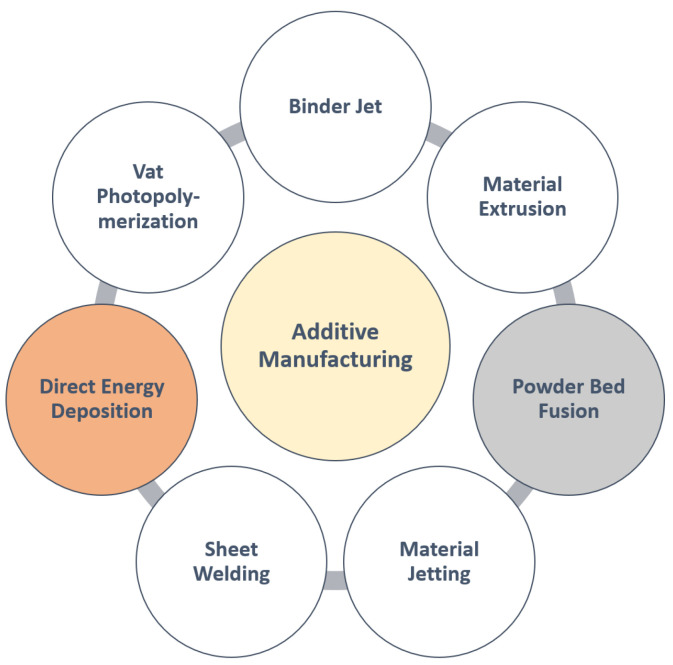
Types of additive manufacturing technology.

According to the American Society for Testing and Materials (ASTM) [2], there are thefollowing seven main types of AM processes: (i) Binder Jetting, which is a process involving selectively depositing a liquid binding agent onto the material; (ii) Material Jetting, which is a process involving depositing droplets of materials; (iii) Material Extrusion or Fused Filament Fabrication, which uses a nozzle to deposit materials; (iv) Direct Energy Deposition (DED) or Direct Manufacturing, which uses a power source to melt metallic material and create the desired part; (v) Sheet Welding, which is a process involving bonding sheets of materials together; (vi) Powder Bed Fusion or Laser Sintering, which is a process using thermal energy to melt material from a powder bed and creating the part; (vii) Vat Photopolymerization, which uses light to cross-link a liquid resin and turn it into a solid. These processes are illustrated in Figure 1.

On the other hand, Metal Additive Manufacturing (MAM) technology is a 3D printing concept used to create a complex metal product from a Computer-Aided Design (CAD) drawing. MAM technologies can lower the cost of creating metallic products, shorten lead time, and eliminate the need for additional processing steps compared to other methods [3,4]. MAM techniques also allow for more flexibility in creating complex geometries and offer better control over the deposition process [4]. As a result, there has been a significant increase in interest in MAM in the last decade. According to a report by Allied Market Research [5], the global MAM market was valued at 2.6 billion in 2021 and is expected to reach 14.1 billion by 2031, growing at a compound annual rate of 18.1% from 2022 to 2031.

**Figure 2:**
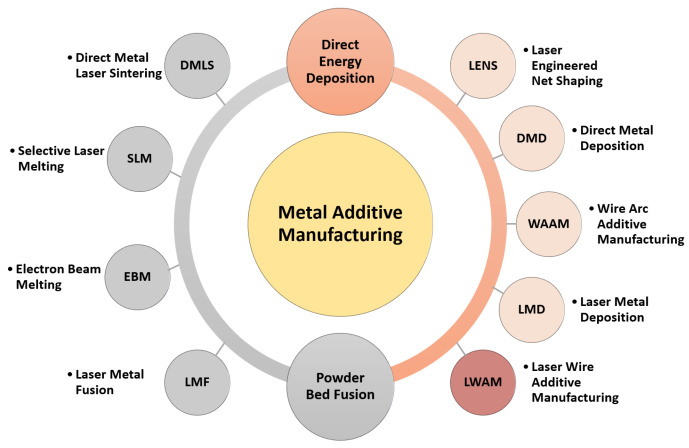
Metal additive manufacturing technologies.

Metal additive manufacturing (MAM) can be divided into two main categories: Powder Bed Fusion (PBF) processes (e.g., [6,7,8]), which use thermal energy to fuse powder selectively, and Direct Energy Deposition (DED) processes (see e.g., [9,10,11]), which use thermal energy to melt a material to form the part [12]. Within the PBF category, there are five main processes: Selective Laser Melting (SLM), Selective Laser Sintering (SLS), Direct Metal Laser Sintering (DMLS), Electron Beam Melting (EBM), and Laser Metal Fusion (LMF). The DED category includes five main processes: Direct Metal Deposition (DMD), Laser Engineered Net Shaping (LENS), Laser Metal Deposition (LMD), Wire Arc Additive Manufacturing (WAAM), and Laser Wire-Feed Additive Manufacturing (LWAM). Figure 2 presents the various MAM techniques. More detailed information on each of these MAM technology types can be found in numerous review articles in the literature, as shown in Table 1.

**Figure 3:**
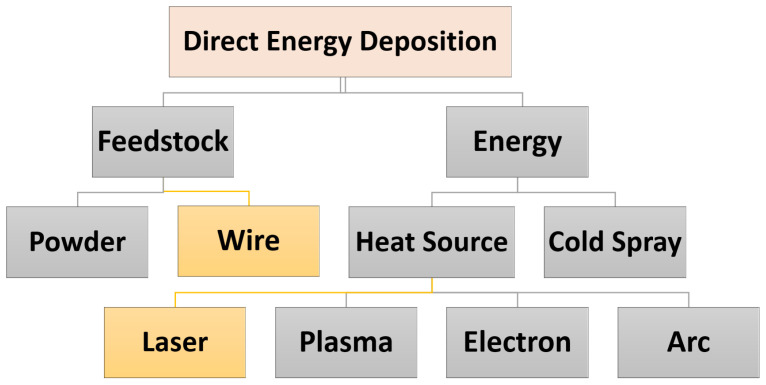
Classification of direct energy deposition.

In MAM, the DED has high energy efficiency flexibility and time-saving, and does not need a special tool to create the metal product, compared to PBF [22]. DED technology can also be divided into two classifications based on the energy source and the feedstock, as illustrated in Figure 3. Metallic deposition processes can use either a powder or wire feedstock. In general, the powder-feed process is more practical for creating small parts with high geometric accuracy, while the wire-feed process is better suited for creating large and complex metal parts. The wire-feed process has a higher deposition rate than the powder-feed process, which is desirable for the fast manufacturing of products. Additionally, the wire-feed process does not require a sealed chamber, unlike the powder-feed process, which must be run in a safe manner to prevent fine metal powder from being dispersed. As a result, the wire-feed process does not have limitations on the size of the products that can be manufactured. According to Ding et al. [50], the wire-feed process has several advantages compared to the powder-feed process. It is a cleaner, more environmentally friendly process and does not expose operators to potential hazards. It also has a higher deposition rate, reaching up to 100% of the wire material. In addition, the wire-feed process is more cost-competitive and more readily available than metal powders. Furthermore, the wire-feed process allows for a larger build volume, with almost no size limitations, as long as the printing device has the capability (see e.g., [19,24,25]). In the state-of-art there are two technologies concerned with wire metal additive manufacturing; i.e., Wire-Arc Additive Manufacturing (WAAM) and Laser Wire-Feed Additive Manufacturing (LWAM).

Wire arc additive manufacturing (WAAM) utilizes a heat source, such as gas tungsten arc welding (GTAW), gas metal arc welding (GMAW), or plasma arc welding (PAW) to fabricate the desired product. The speed at which material is deposited during WAAM varies, based on the material being used, with the highest rates achieved using a combination of GMAW and metal wires. GMAW, also known as metal inert gas (MIG)/metal active gas (MAG) welding, uses electrodes and a heat source to create the toolpath with minimal interference. However, GMAW has a restricted build volume, due to the large amount of heat and melt pool it generates. WAAM has some drawbacks compared to LWAM, including the need for specialized equipment and specific safety measures for handling the materials, which can affect the quality of the final product and pose an environmental risk. WAAM also has a lower deposition rate, which leads to longer product creation times [51].

**Figure 4:**
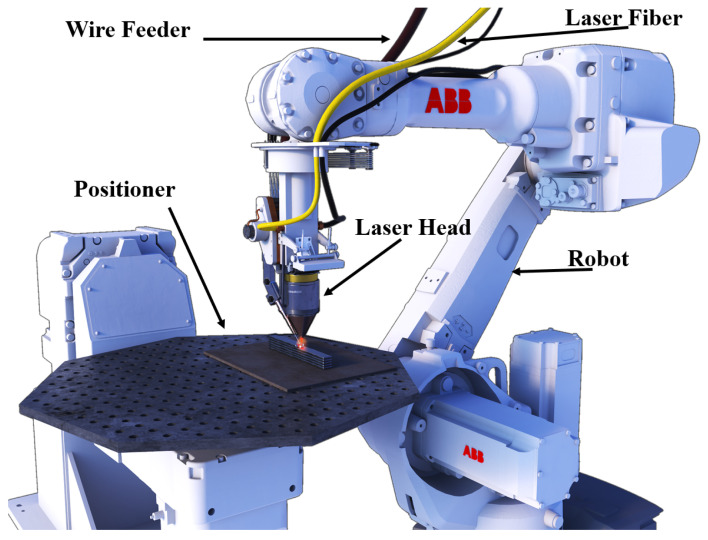
Process setup of laser wire-feed metal additive manufacturing.

This paper examines the Laser Wire-Feed Metal Additive Manufacturing (LWAM) technique, which is a variation of the Directed Energy Deposition (DED) method. LWAM uses a high-power laser to create a pool of molten metal, which is then deposited on a substrate and built up, layer by layer, using a moving head to create a solid object. The LWAM system typically includes a six-axis robot, a laser head, an automatic wire-feed system, and, sometimes, a positioning table, as illustrated in Figure 4. In order to prevent contamination during the deposition process, the LWAM system often uses a shroud of shielding gas. When working with reactive metals, it is necessary to use a fully inert chamber with a large amount of gas and sufficient time to achieve the desired oxygen levels [29].

The LWAM process allows for the creation of complex products and offers the ability to work with complex shapes. It also allows for wider path widths, which enables higher deposition rates and can lead to lower manufacturing costs and faster production times, particularly for large parts, compared to other methods that require post-processing. Additionally, the LWAM system uses high power density and flexible lasers to provide good control in the manufacturing process, resulting in parts with near-net shape features and better metallurgical qualities. This leads to cost savings and a high level of design freedom. The products produced through the LWAM process have strong bonding structures and nearly zero porosity [30].

**Figure 5:**
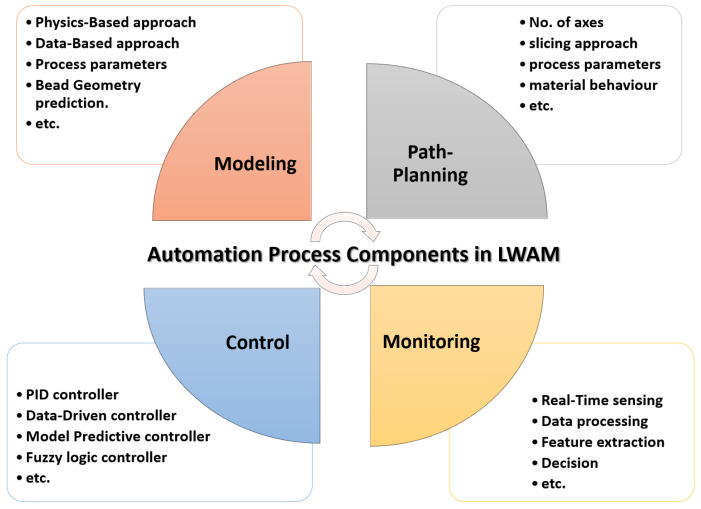
Automation process components of laser wire-feed metal additive manufacturing.

Despite all the advantages of LWAM technology, it is still in a mature state and its integration is still in progress in the industrial sector. Several issues and challenges still exist in LWAM, including low quality of printed layers, accumulative errors, and the difficulty and expensive design of proper monitoring and control systems. Therefore, this work aims to provide a comprehensive review of the state-of-art on LWAM to obtain a clear vision of the research status and advancements and to discuss the future perspectives of this technology. Compared with the previous reviews of MAM, presented in Table 1, this review paper presents the research progress in the primary process technologies of LWAM, including path planning, modeling, monitoring, and control systems, as depicted in Figure 5.

To this end, the rest of the paper is organized as follows. Section 2 presents a review of various parametric and physics-based modeling using different methodologies and techniques related to LWAM. Section 3 provides an in-depth review of the monitoring and control systems that are used in LWAM. Section 4 is devoted to the current research and challenges on path planning for robot-assisted 3D-Printing using LWAM. Finally, Section 5 and Section 6 summarize the key findings of the review on LWAM technology, identify major themes and trends, and highlight potential research gaps. Possible areas for future research to further advance the LWAM technology are also suggested.

## 2. Modeling and Parametric Study of the Bead Deposition in LWAM

**Figure 6:**
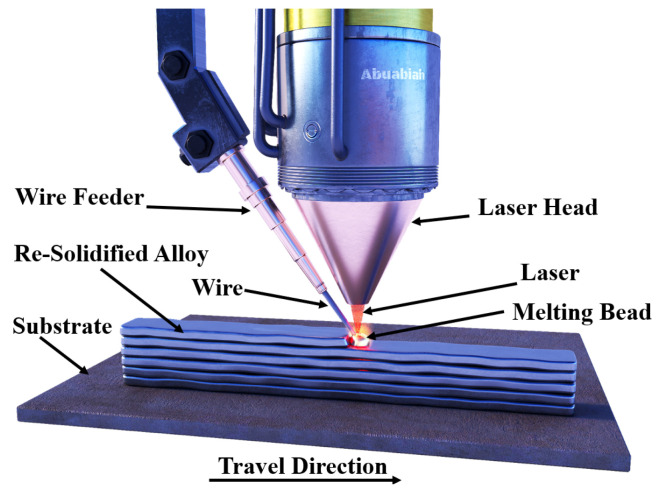
Diagrammatic representation of the LWAM’s bead deposition mechanism.

LWAM utilizes a metal wire feedstock and a laser to create a molten pool. A protective shield of gas is used to keep the material being deposited uncontaminated. The process involves melting a wire with an energy source to form a liquid bead, which is then added, layer by layer, to create the final product, as depicted in Figure 6.

**Figure 7:**
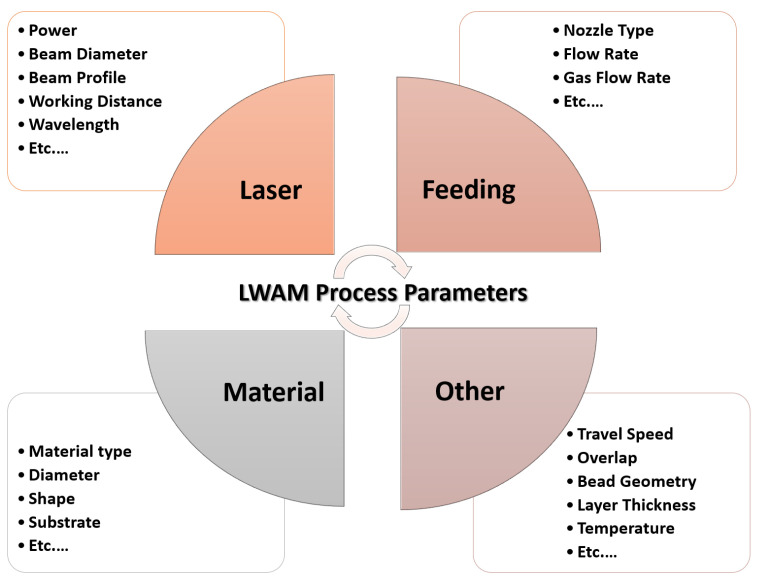
Process parameters of laser wire-feed metal additive manufacturing.

In the process of LWAM, it is important to consider the process planning, deposition techniques, and combination of parameters. A major challenge in this wire-based approach is ensuring that the process remains stable, which can be influenced by factors such as the orientation and speed of the wire feeding and the direction of deposition. Other important parameters, such as power, travel speed, and temperature, also impact the formation of the melt pool and the geometric accuracy of the final product. The most significant parameters in the LWAM process are depicted in Figure 7. Since the LWAM process involves a wide range of parameters, changes to variables, like the cooling rate, power, or speed, can alter the shape of the melt pool and affect the quality and integrity of the finished product [52,53].

Reliable sensing and control techniques are required to provide a steady deposition process. However, one of the main challenges of designing a suitable controller is providing an accurate model of the bead geometry deposition in LWAM and understanding the system’s behavior and characteristics. The literature discusses a number of techniques, including parametric study, and computational, empirical, simulation, and physical-based models of LWAM. For example, Mortello and Casalino [54] observed the weld pool dynamics and metal transfer mode in real-time for wire laser additive manufacturing. The proposed system enabled smooth and regular metal deposition, where the geometrical and microstructural properties for 15 layers of Titanium alloy wall were satisfactory. N. Mbodj et al. [55] proposed a layer geometry prediction model using machine learning strategies. They also studied the effect of laser power, wire feed rate and travel speed on bead geometry using a neural network model. A prediction model, with low errors and good layer deposition, was derived. Ding et al. [56] studied the effect of ambient pressure on the bead shape of 4043 Al alloy manufactured by laser coaxial wire feeding additive manufacturing for single-layer objects. The study found that decreasing the ambient pressure to 1 Pa resulted in a wider and smoother deposited bead, with a lower layer height and smaller contact angle. This was due to the reduced influence of the plasma plume on laser loss at low ambient pressure, leading to a larger molten pool and less oxide production, which reduced surface tension and allowed the melt to spread more easily.

Furthermore, W. Huang et al. [57] studied the relationship between process parameters and bead geometry for aluminum and its alloys manufactured by LWAM technology. They established a second-order polynomial equation to relate the LWAM process parameters and layer geometry characteristics, and found that the highest deposition weight was achieved when the wire feeding angle was at 45°. They also studied the effect of travel speed and laser power on the deposition width and found that laser power was more significant than travel speed in this manner. Zapata et al. [58] studied the effect of process parameters on bead and layer geometry for coaxial laser wire-based metal deposition using aluminum (AlMg4,5MnZr) and stainless steel (AISI 316L) wires. The research classified the resulting beads as stubbing, dripping, or good beads. Insufficient energy input could result in beads with short pieces of unmolten wire (stubbing), while using excessive energy, an insufficient wire speed, or an inadequate focal offset could cause the wire to melt before it reached the substrate surface (dripping). The authors studied the process parameters that impacted the bead geometry and created map charts for this purpose. They found that the relationship between process parameters and bead height and width could be approximated by linear models.

On the other hand, Kumar et al. [59] proposed a simulation tool for choosing the best parameters and melt pool conditions. With changing laser power and power density, the provided simulation tool recognized the heat transfer and fluid flow behavior of wire feed additive manufacturing using a transient 3D model. The model was written in Python, and tests were carried out with a 1 kW Gaussian beam fibre laser. On Ti-6Al-4V alloy, the impact of laser exposure on the scanned and deposited profile was revealed. This model can accurately envisage the temperature profile and the profile of a solidified metal, according to a comparison of simulation and experimental results. The model is able to determine the best input parameters, based on the properties of the materials. Guo et al. [60] proposed a multi-channel, three-dimensional transient numerical model of laser wire-feed additive manufacturing using 5A06 aluminum. With the aid of a mass source term and a three-dimensional conical Gaussian heat source. The numerical simulation in the Fluent program was implemented for continuous wire feeding. Under various lap ratios, the geometric morphological properties of multi-channel laser wire-feed additive manufacturing were investigated. Using geometric morphology as the assessment index led to finding the best lap ratio. This study could also help with multi-channel laser wire-feed additive manufacturing lap distance selection, enhance the geometric shape of the parts deposited, and is important for the prevention of faults in a multi-channel deposition.

To investigate how process factors affect deposition, Fetni et al. [61] created an empirical model using the finite element method. The thermal history and melt pool dimension progression of 316L stainless steel, reinforced with tungsten carbides, were investigated. Utilizing experimental findings from light optical, scanning electron, and thermocouple records, the experimental analysis was connected with the numerical outcomes. Corbin et al. conducted [62] a single bead geometry empirical model with different process parameters. Using an optical profilometer, linear regression was used to fit the data that had been gathered. The bead height, bead width, and angle of repose were the response variables. The model demonstrated the connection between the process parameter and the bead geometry. Nie et al. [63] conducted a laser hot-wire (LHW) experiment to deposit material at a rate of 3.46 kg per hour by using less than 10 kW of laser power. To track the temperature changes during the deposition, four thermocouples were integrated into the substrate. The findings demonstrated that temperature changed periodically as the layers were deposited. The substrate’s distortion was measured concurrently. The maximum distortion of the substrate increased gradually throughout the deposition. In this article, a thermal–mechanical FEA model of the LHW process was used. The calculations were done for the temperature, stress and strain, and distortion. Based on the temperature parameter, the reason for the change in microstructure was explained. Last but not least, a numerical analytic approach was used to map the processing window of stable hot-wire deposition.

According to Hatala et al. [64] the use of nickel aluminum bronze (NAB) alloys in large-scale MAM processes for maritime applications is growing in popularity. In order to forecast part distortion, this study outlined the creation and assessment of a thermo-mechanical simulation tool for NAB’s LWAM. The main findings of this study were the following: (i) experimental measurement of temperature-dependent material properties of NAB alloys; (ii) development and evaluation of heat-source model simplification to account for hot wire and oscillating laser beam; (iii) thermo-mechanical analysis and assessment of the impact of temperature-dependent, versus constant material, properties on distortion prediction. Furthermore, by creating single- and multi-layered tracks, Huang et al. [57] investigated the laser-beam deposition of Al alloy 5A06 wire. Prior to examining the effects of the primary process variables, such as laser power, traverse speed, and wire feed rate, on the geometrical properties of the deposited, the effect of the wire feeding direction and angle were examined. The experimental specimens were prepared for optical microscopy observation by being cut, ground, and polished. The measured data was then compared to the projected values predicted by the mathematical models. Images of the weld pool and droplet transfer, which demonstrated the stability of the welding process, were taken, using a high-speed camera system. Finally, utilizing the adjusted process settings, a thin-wall component was created without any discernible pores, cavities, or flaws.

Using information gleaned from experimental data, Liu et al. [65] proposed a multi-property integrated design framework to facilitate quality control of bead surface finish, geometry, and microstructural properties. The relationships between the printed bead process, microstructure, geometry, and process feature importance scores were examined. To create the ideal processing window, the features of the microstructures, geometry, and overall bead quality were simultaneously optimized. In particular, the desired property thresholds were established experimentally, and machine learning models predicted and visualized, in a 3D contour space, the microstructures and geometries of printed beads under various process combinations. By removing undesirable process candidates from a process searching space using these predictions, the process parameters were then optimized. The verification findings demonstrated that this framework’s optimized process parameterizations could speed up the production of materials with the desired bead multi-performance. Finally, Liu et al. [66] gathered an experimental dataset that spans a variety of process variables and output characteristics, including overall bead quality, geometric shape, and microstructures. Then, by using data-driven machine learning models, the expected connections between process, geometry, and microstructure can be captured. A three-dimensional contour map is used to visualize the properties of printed beads over a wide range of processing space. For the purpose of enhancing quality during manufacturing processes, the insights and effects of process variables on bead morphology, geometric, and microstructural features were fully examined.

Table 2 provides a comprehensive summary of the main findings regarding parametric modeling approaches used in Laser Wire Additive Manufacturing (LWAM). It outlines the various mathematical approaches used, such as mathematical models, analytical models, and regression models, and highlights the advantages and limitations of each approach. The table also summarizes the key features of each parametric modeling approach, such as the input parameters, the output parameters, and the modeling software used (if any).

## 3. Monitoring and Control Systems of LWAM

As previously mentioned, the LWAM process involves using a high-power laser to create a molten metal pool. The pool is deposited onto the substrate and then built up, layer by layer, as the robot head moves, forming a solid object. To ensure the stability of the bead deposition process, LWAM requires reliable sensing and control approaches. The laser wire-based technique is sensitive to various deposition parameters, such as power, travel speed, and temperature, which are crucial for the formation of the melt pool and the geometrical accuracy of the final product.

**Figure 8:**
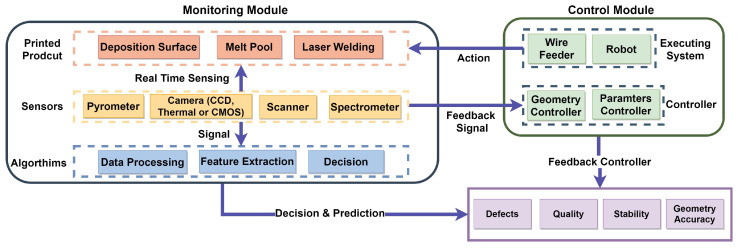
Framework of process monitoring and control in LWAM [24].

The general process monitoring and control framework of the laser-metal additive manufacturing system is shown in Figure 8. This framework was adopted with modifications from Xia et al. [24]. The overall system is made up of two parts: a monitoring module, that uses multiple sensors to detect problems, and a control module, that adjusts certain aspects of the process in order to improve the quality and accuracy of the finished parts. The monitoring module is in charge of collecting data from various sensors and alerting the system to any issues that may arise. The control module, on the other hand, works to regulate certain factors related to the shape of the beads and the process parameters in order to produce better results.

With the development of sensing devices and control systems, several works have been developed in the past years for monitoring and controlling the deposition in metal additive manufacturing in LWAM. More specifically, Heralic et al. [70] developed a monitoring system integrated with the robot for automatic in-process height control of the deposition. The monitoring system includes a camera and a 3D scanner to enable online visual feedback for both the process and the deposited layer’s topological profile. A 3D scanner is used to capture images of each layer as it is being deposited. These images provide a detailed understanding of any disruptions or abnormalities that may occur during the process, allowing the system to be controlled more effectively. An iterative learning algorithm controller was developed to adjust the wire-feed rate based on the scanned data of the part being deposited. This controller was able to detect changes in the material as it was being deposited and to make adjustments to maintain a smooth, flat surface without any prior knowledge of the disruptions that might occur. The results showed that the developed controller was able to effectively compensate for local changes and produce a more consistent final product.

Recently, Garmendia et al. [71], developed a methodology for measuring and controlling the deposition height, layer by layer, using a structured light-based 3D scanning technology. To improve the accuracy of the printed workpiece, the layer height is first adjusted based on the scanned height profile of the workpiece and the reference CAD geometry. The developed controller then adjusts the local height deviations by changing the scanning speed, which adjusts the feed rate in areas that are too high or too low. The proposed controller was tested and found to be effective in correcting local defects within a layer and producing more consistent, planar layers after a certain number of layers had been deposited. Furthermore, Garmendia et al. [72] proposed a closed-loop geometric control to adjust the height deposition trajectories during the metal deposition in different building stages using an adaptive Computer-Aided Manufacturing control strategy. To achieve a closed-loop feedback controller a measuring system, that consists of a structured light scanner to obtain the height profile of the deposited layer, was used. The proposed control strategy was able to stabilize the process deposition as long as minor deviations between the last layer height and the laser focusing point were properly monitored.

On the other hand, Xiong et al. [73] proposed a new type of controller that uses a single neuron to intelligently adjust the width of each layer in wire and arc additive manufacturing processes. The controller takes the travel speed as input and adjusts the output and the layer width, accordingly. To test the effectiveness of the developed controller, a 13-layer thin-walled structure was deposited with varying layer widths. The authors used a second-order Hammerstein model to describe the relationship between layer width and travel speed, and, based on this model, they developed the intelligent controller. The results showed that the controller was able to maintain a maximum absolute error of 0.5 mm between the measured and desired layer widths, and was effective in increasing process stability within the optimal layer width range of 6 to 9 mm. Becker et al. [74] developed a closed-loop process control to print an accurate small wall-thickness metal product. The controller was designed to track the height and subtractive processes using internal and external longitudinal turning with varied parameters. Using optical coherence tomography (OCT) distance measurement the designed closed-loop controller was able to track bead height, through the control of the wire feed rate, to obtain a more uniform layer structure. To improve the surface roughness subtractive machining operation was also adapted. The results showed that a cylinder of thickness 2.3 mm could be printed using the designed controller with only 9.67% of the material having to be removed to obtain the final shape.

Liu et al. [75] proposed a model predictive controller (MPC) for controlling the size of the melt pool in a laser-based manufacturing process. A first-order transfer function model, with a time delay and laser power as the manipulated variable, was used to represent the system, which was then converted into a non-minimal state space representation with all measurable state variables. The melt pool size was measured using an inexpensive near-infrared monochrome camera. The experimental results demonstrated that the proposed MPC was able to maintain the melt pool size at the set point under various process conditions, with minimal overshoot and a short settling time. A model predictive controller was also used in the work of Mbodj et al. [76] to maintain the layer height trajectory constant by taking into consideration different constraints faced in the LWAM. The authors first developed a model that described the relationship between the layer height and various process inputs, such as process parameters, thermal history, and material properties. This model was based on physical principles and took into account the way that the bead geometry was affected by the inputs. Next, they designed a model predictive control (MPC) controller to follow a predetermined reference trajectory for the layer height by adjusting the temperature input, while keeping other parameters within their normal operating range. The results showed that the MPC controller was able to effectively track the reference signal and maintain a constant bead height.

Gibson et al. [77] proposed a real-time closed-loop melt pool size (MPS) control using laser power as the manipulated variable and a thermal camera for imaging the melt pool. To enable the control of either average laser power alone, or of average melt pool size, a second controller that modulates the deposition rate and print speed on a per-layer basis was developed. A third controller was also proposed which combines the previously mentioned controllers by modulating the laser power to control MPS in real-time, and the nominal deposition rate and the print speed were manipulated to control average laser power on a per-layer basis. The results showed that the first MPS control architecture yielded a fine, local control of bead geometry in comparison to the second controller, while the third controller provided a reduction in the printing time, and the printed quality was comparable to the first controller. Furthermore, Gibson et al. [78] developed a closed-loop control system for managing the size of the melt pool in LWAM. The system adjusts the setpoint at certain trigger points in order to make specific changes to the process, such as modifying the melt pool size. This allows the system to automatically achieve consistent property modifications, even when the thermal conditions change during the construction of the final product. The proposed controller was tested by printing a double-bead wall to demonstrate its ability to make local process changes and print an extra-toolpath geometry beyond the normal toolpath. The controller was successful in these tasks.

Another concept for process monitoring in LWAM using IR temperature measurement and optical coherence tomography, as well as a multi-variable closed-loop process control, was proposed by Bernauer et al. [79]. The proposed control architecture consists of the bead height and the melt pool temperature as the controlled variables, while the wire feed rate and the laser power were the manipulated variables. It is worth mentioning that the authors only provided the concept and the framework without real implementation. Therefore, there is not yet experimental evidence to confirm the effectiveness of the proposed framework. However, the same team, Bernauer et al. [80], proposed a PI closed-loop controller to track a predefined temperature reference signal to lower the defects of the final product. The controller was tuned based on an identified dynamic model system that described the relationship between the temperature and the laser power. Lastly, the team of Bernauer et al. proposed in [81] a closed-loop control system to regulate the melt pool temperature in a pre-defined way to examine the influence on the process stability and the resulting bead geometry characteristics. To do this, a process window that contained the temperatures as a reference signal was determined to be tracked in a stable manner. Then, the correlations between the bead characteristics and temperature were evaluated with the process window.

Finally, Chen et al. [82] proposed a control strategy that uses data-driven machine-learning techniques to adjust the laser voltage, based on the size of the melt pool, which is monitored using a special unit. The control strategy is based on the virtual-reference feedback tuning (VRFT) algorithm and is used to automatically tune a PID controller. One of the benefits of this proposed controller is that it does not require a detailed system identification or physical model of the process, and it can adapt to different materials, part shapes, tool paths, and process parameters. The results showed that the controller was able to produce metal parts with improved geometric accuracy, less fluctuation, and better consistency compared to uncontrolled and conventional PID controllers.

In summary, Table 3 provides a list of monitoring systems and control algorithms that are commonly used in LWAM. Some of the key features highlighted in the table include the type of sensor technology employed, such as infrared cameras, 3D cameras, profilometer, or pyrometer sensors, and outline the specific control algorithms used in LWAM, which include both feedback and feedforward control systems.

## 4. Path Planning for Robot-Assisted 3D-Printing Using LWAM

The workflow of 3D printing starts from geometry generation using CAD software. This is followed by converting it into an STL file. Currently, most software that is used takes this STL file and converts it into a G-code, which contains a series of orders for the 3D printer to follow. This step is widely known as slicing. Examples of such software are Cura, Slic3r, OctoPrint, etc. Although these machines can be structured differently, such as Cartesian and Delta, the basic motion design is based on a combination of 3-axis linear movements in the X, Y and Z axes. Such slicers mainly slice the 3D geometry into layers perpendicular to the printing direction. Afterwards, it generates a path for the moving head for printing the perimeters and the infill. In some printing technologies, such as extrusion-based, it also assigns values for the material flow during the printing process.

The tool path generation techniques from the above-mentioned software are based on 3-axis movement. However, new research is emerging that utilizes more than 3-axis machines. This can be seen when a printing head is mounted on an industrial robot with 6 axes and, in some cases, coupled with a 2 axes tilting and rotating table. Such higher degrees of freedom brings more flexibility in producing the required objects when it comes to reducing the need for support materials, for example. However, the main challenge is to provide algorithms that can generate a path for the tool independent of the required shape complexity. This is done by taking into consideration the constraints in the robot’s movement, aka the robot’s workspace. Additionally, it is necessary to take into consideration the best parameters (head orientation, material flow, energy, etc.) for the printing technology used.

Over the past years, several contributions have emerged in developing algorithms for tool path generation to control the movement of the printing head and, in some cases, the material flow during 3D printing. This was done considering the utilization of 3+ axes machines. Ding et al. [85] developed a method based on the decomposition of the required geometry into subvolumes using a curvature-based volume decomposition method. Then, the topological information is used to merge the volumes into ordered groups and generate a toolpath for each group. McPherson and Zhou [86] proposed a method for generating the path for mobile printing robots. The idea was to work with more than one printing robot simultaneously without them interfering with each other. This was done by the development of a chunk-based slicing method that divides the required geometry into parts. Each one is assigned to a printing robot in order to achieve synchronized printing. Munasinghe and Paul [87] worked on a method for slicing radial objects using concentric ray lines. This can be an interesting method for printing helical-shaped objects, such as extruders or spiral conveyers. Their method was proposed for large-scale AM techniques to avoid using support materials during printing. Schmitz et al. [88] solved the slicing problem starting with the robot’s kinematics. In their slicing algorithm, the tool path was generated taking into consideration the robot’s joint velocity limits. This method showed improvement in the robot’s workspace coverage compared with other slicing methods.

Other researchers worked more specifically using one 3D printing technology to provide a proof of concept for their proposed slicing algorithm. McCaw and Cuan-Urquizo [89] developed a slicing approach based on Bézier surfaces of the arbitrary order method. The aim of their work was to produce a path for the printing head for depositing material on a non-planer surface. It was possible to fabricate lattice structures using a parent non-planer surface. In this way, there was no need for producing a support structure to fabricate the required lattice structure. They used a conventional 3-axis 3D printer and a polymeric filament to showcase their slicing approach. Zhao et al. [90,91] developed a mixed-layer adaptive slicing method. The main idea was to use planer and non-planer slicing depending on each sub-domain of the geometry. They used a 6-axis robot to test their approach using a polymeric extrusion printing head. Yigit and Lazoglu [92] introduced a spherical slicing approach. In their method, the geometry is divided into a set of spherical layers that, when combined, produce the final shape. The tool path is then generated based on each layer. A 6-axis robot was used to test the approach using a polymeric extrusion head. Kontovourkis and Tryfonos [93] developed a parametric-integrated approach for path generation of the printing head. An industrial 6-axis robot was used with a clay extrusion head. Chalvin et al. [94] developed a layer-by-layer slicing approach, taking into account the trajectory optimization based on its limitation during printing. This approach showed improved 3D printed part accuracy by increasing the efficiency of the 3D printing process. For the proof of concept, a 6-axis robot was used with a polymeric extrusion head. Zhao et al. [95] focused on slicing revolving parts. They introduced a ray-based slicing approach. This method, along with helical path planning, was used to cover non-uniform path spacing between adjacent paths in the same layer. This method was tested using a rotary 3D printer with degrees of freedom (2 translations and 2 rotations) using a polymeric extrusion head.

Moreover, other researchers used wire arc additive manufacturing to test their slicing approaches. Zhao et al. [96] developed a unit block-based planning approach. They aimed at slicing geometries with varying layer thicknesses. The unit block is dependent on the process parameters, such as deposition rate and geometric shape. Their approach resulted in improving surface waviness, deposition rate and material utilization. For their proof of concept, they produced parts from Aluminum using a 6-axis robot. Hu et al. [97] developed a slicing approach that ensures the welding takes place on a horizontal plane. This attempted to improve the bead uniformity by removing the gravity effect. In their approach, the geometry was divided into subregions and a path for the tool was calculated for each one. To test their approach, prototypes from steel were realized.

In recent years, laser wire-feed technology has been adopted in additive manufacturing and many researchers have been using this technology to test their slicing algorithms. In the following examples, researchers used a 6-axis industrial robot coupled with a 2-axis (tilt and rotary) positioning table. Several alloys were explored, such as stainless steel, mild steel, and Ti-6Al-4V. Ding et al. [98] worked on a hybrid slicing technique. This utilises the extra degrees of freedom offered by the positioning table. This approach recognizes the overhanging regions and revolves around the printed object to achieve a planer printing path. This improved the accuracy of the produced structure with the CAD model. Moreover, they worked on another approach [99], in which a Matlab code was developed to generate a tool path for the robot. This path took into consideration volume slicing, contour filling, track trimming/elongation, stair-step effect compensation and post-processing. Gibson et al. [100] also attempted to generate a tool path with consistent deposition orientation relative to gravity. Moreover, they took into consideration the ability of the path to produce an overhang structure without any need for support. They used an alternating tool path direction approach and the manipulation of the CAD model surface normal vectors. Finally, Mbodj et al. [101] proposed a method that employs the use of a parametric design approach to simulate and fabricate 3D metallic objects using the LWAM process. It involves the formation of patterns and the assignment of targets for the robot, considering the various process requirements of LWAM and the robot system. This leads to the development of an adaptive robot tool path for an optimal deposition process. The technique was validated by printing metal objects, such as a wall, a cylinder, and a complex shape, both in simulation and experimentally. The results indicated that the approach is feasible and adaptable, and can enhance 3D metallic printing in the LWAM process.

Table 4 provides a comprehensive summary of different path-planning approaches used in Manufacturing Additive Manufacturing (MAM), including various printing setups, such as mobile robots, 6-axis robots, and multi-axis robots. The table outlines various path-planning approaches, including geometric-based and slicing-based methods, which are employed to generate the toolpath. Additionally, the table offers insight into how different printing setups impact the path-planning process, as each setup may require specific path-planning techniques and algorithms.

## 5. Discussion

AM technologies are showing promising capacities in many fields. Metal AM technologies, such as LWAM, are an emerging field. Many researchers are working intensively to improve this process’s capabilities and to overcome its limitations. Therefore, this study presents a thorough overview of the current state-of-the-art for LWAM technology by analyzing the recent research advancements in key areas of LWAM, including path planning, parametric study and modeling, monitoring, and control systems.

**Figure 9:**
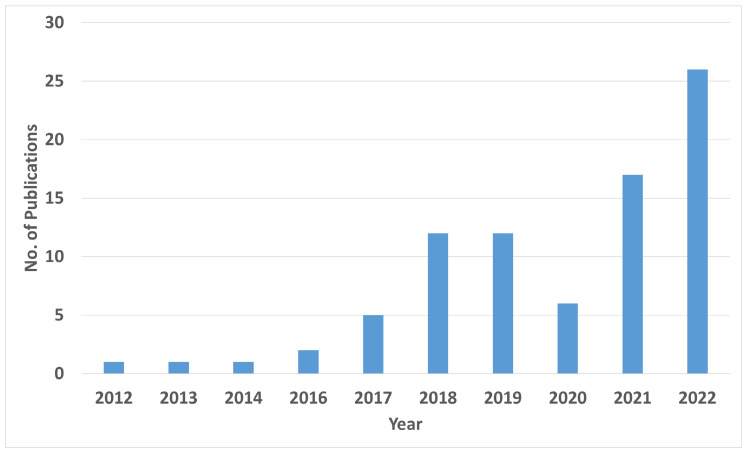
Number of articles per year related to LWAM technology. This search was conducted via WebOfScience on 16th February, 2023, using the following search criteria: “laser wire additive manufacturing” (Topic) or “laser wire-feed additive manufacturing” (Topic) or “laser-based direct energy deposition” (Topic) or “Wire-feed Laser Additive Manufacturing” (Topic) or “Laser Metal Deposition with Coaxial Wire Feeding” (Topic) or “laser-wire directed energy deposition” (Topic) or “laser metal-wire deposition” (Topic) or “laser metal deposition with wire” (Topic).

Figure 9 depicts the increasing trend of research interest in LWAM over the period from 2012 to 2022. The data for this figure was obtained through a search on WebOfScience, using specific search criteria related to Laser Wire Additive Manufacturing. The results showed an almost steady rise in the number of publications on the topic over the years, indicating a growing interest in this area of research. Laser wire-feed additive manufacturing has become a popular alternative to traditional methods of metal additive manufacturing, offering improved efficiency, precision, and versatility.

According to the state-of-the-art related to bead geometry modeling and parametric study (provided in Section 2 and summarized in Table 2) it can be concluded that the computational technique for finding an appropriate mathematical model in LWAM has several limitations. This is mainly due to the complex nature of partial differential equations of the system with many process parameters (e.g., laser power, wire feed rate, travel speed, temperature, layer thickness, working distance, etc.). The disadvantages of numerical and empirical approaches in LWAM include the requirement for several experimental tests in order to gather response data and to create a well-defined model. Large data management and lengthy computation times are the primary drawbacks of empirical, computational, and numerical models in all the publications listed. Additionally, the models were too complex and constrained to be used in the development of control systems. As a result, an accurate model for LWAM is still needed for process optimization and control design. A solution window for such a problem could be the use of a Machine Learning (ML) algorithm. ML has proved its efficiency in many areas where physical models are hard to determine. Several studies have recently tried to use ML in MAM (see e.g., [102,103,104,105,106]) but still, there is a lack of use of ML in the LWAM for bead geometry prediction based on the process parameters, and further study is needed.

On the other hand, in this review paper, the research efforts conducted in the area of monitoring and control systems in LWAM were discussed (see Section 3 and Table 3). The research on monitoring systems is mainly conducted to measure the deposition temperature, using a pyrometer/thermal camera, bead height and width, using a 3D scanner/profilometer, laser power, using pyroelectric, and wire feed rate, using a tachometer. So far, image processing using a CCD camera is the main signal used for monitoring the geometric accuracy and temperature distribution in LWAM. Scholars have also explored various control algorithms and techniques to improve the stability and to enhance the surface quality of the printed product using LWAM technology. These techniques can be varied by using, for example, a classical controller design, such as PID and MPC, or by using data-driven controller algorithms that depend on input/output measurements, or, finally, by using real-time tuning controllers that use more intelligent algorithms, such as the iterative learning controller method. However, in terms of geometric accuracy, the surface quality or ability for monitoring the vast variety of process parameters in LWAM still needs to be improved. More advanced monitoring systems using artificial or multisensor information fusion are advisable. Designing proper intelligent controllers with more robustness to process parameter changes should be considered the future of R&D in the field of LWAM to print large metal products with improvements in surface quality and accuracy.

Finally, the toolpath generation for 3D printing complex shapes using 3+ axes equipment is not an easy task. However, researchers are keen on providing the optimum solution for this problem (see e.g., Section 4 and the summary Table 4). There are many aspects to consider which can be on different levels, namely, geometry, processing parameters, and the object’s final properties. On the geometry level, these extra degrees of freedom open the doors for more efficient 3D printing by producing complex parts with minimum support for the overhanging regions. Nevertheless, this comes with the price of more complexity during the analyzing of the CAD models. Dividing the geometry into regions/volumes and adaptive layer thicknesses to allow easier planning for the toolpath can be an innovative solution. However, it is very important to have a good understanding of the resulting mechanical properties of the produced part, especially in regions where two volumes merge. The processing parameters level is quite a complex problem, as it requires taking into consideration the physics of the printing technology and coupling this understanding with the shape being produced. Some researchers attempted to omit the effect of gravity in order to produce a uniform bead (material deposition) as much as possible. However, still, the research is limited when it comes to coupling the process parameters, such as energy, feed rate, etc., with the path planning and slicing process. Finally, the object’s final properties are very important, as they allow the production of functional parts with acceptable quality. The main issue to consider during slicing is the behavior of the material after printing. Shrinkage and internal stresses may cause the final geometry to deviate from the initial design. This should be taken into consideration during the slicing process. On the other hand, the toolpath generated should assure optimum mechanical quality with predictable material microstructure. The research focusing on this topic is very limited and more work should be done in this field.

## 6. Conclusions

Scientific and economic interest in LWAM has been increasing rapidly in the last few years. This interest is shown by the significant number of studies conducted so far in the field of LWAM (see Figure 9). Our review focused mainly on aspects related to this technology, such as modeling and parametric study of the bead deposition, monitoring and control systems used in this process and path planning for robot-assisted 3D printing. The demand from industry has pushed the scientific community to work on the current limitations existing in LWAM technology. Therefore, researchers have adopted various methodologies, such as using ML for bead geometry prediction based on the process parameters. However, further studies are needed. Another important aspect is the continuous monitoring of the product’s quality. This has been tackled by applying multi-sensor information fusion and designing proper intelligent controllers. Additionally, the toolpath generation techniques, especially when using industrial robots with higher degrees of freedom, are still a bottleneck in advancing this technology.

## Figures and Tables

**Table 1:** Review articles on MAM technology.

MAM	Technology	Reference
Direct Energy Deposition	Laser Engineered Net Shaping	[13,14,15]
	Direct Metal Deposition	[16,17,18]
	Laser Metal Deposition	[19,20,21]
	Wire Arc AM	[22,23,24,25,26,27,28,29]
	Laser Wire-Feed AM	[30]
Powder Bed Fusion	Direct Metal Laser Sintering	[31,32,33,34]
	Electron Beam Melting	[35,36,37,38,39,40]
	Laser Metal Fusion	[41,42,43,44]
	Selective Laser Melting	[45,46,47,48,49]

**Table 2:** Summary of main findings regarding parametric modeling approaches in LWAM.

Ref.	Year	Findings/Model
[61]	2016	The Finite Element Method (FEM) was used to predict the thermal history and the change in melt pool dimensions during the Directed Energy Deposition of a new composite coating.
[59]	2021	A numerical 3D model of wire feed additive manufacturing was developed using Python, incorporating the effects of heat, fluid flow and laser exposure on the scanned and deposited profile of a Ti-6Al-4V alloy, and tested with a 1 kW Gaussian beam fibre laser.
[63]	2021	An experiment was conducted to deposit material at 3.46 kg/h using a low-powered laser. A numerical simulation was used to study the temperature, stress, strain fields, and distortion. The findings showed that there are limits to stable deposition and high residual stress at the junction between the deposit and the substrate.
[64]	2021	This article performed a thermo-mechanical analysis of the LHW deposit of NAB alloys. The simulation results showed that if constant material properties were used instead, the predicted displacement at the free end of the substrate would be in the opposite direction and that there would be a difference in the predicted vertical displacement if temperature-dependent material properties were measured from the test specimens produced using a DED technique other than LHW.
[57]	2022	This study examined the deposition of Al alloy 5A06 wire using a laser beam, capturing images of the weld pool and droplet transfer with a high-speed camera. It was found that the angle of the wire feed is important for absorptivity, and a mathematical equation was developed to relate LWAM process parameters to the characteristics of the track layers using a second-order polynomial.
[67]	2022	A model was used to predict the temperature of the wire tip during the dynamic preheating process, with and without resistance preheating. It was found that bead formation in the LHWD process is significantly influenced by the wire transfer mode and the stability and efficiency of low-resistivity aluminum-alloy wire LHWD were demonstrated through experimental validation. Analytical models were developed to calculate the preheating temperature of the wire tip without laser melting.
[65]	2022	The framework was tested using Ti6Al4V beads printed on an LWAM system to assess in-process parameters and post-process property characteristics. The results showed that it is an effective tool for optimizing the design of single-layer bead materials using a wire-feed laser AM system, and is expected to have a broader impact in the future as it is also applicable to other multi-functional materials and other AM methods, such as laser powder bed fusion AM.
[68]	2022	High Rate Deposition Directed Energy Deposition (HRP-DED) is a technique that increases both mass and energy input to improve production efficiency, and is widely used to fabricate nickel-based superalloys. The development of HRP-DED is closely related to nozzle parameters, and the maximum deposition rate is 1750 cm^3^/h. The mechanical properties of alloys produced using HRP-DED have been primarily studied for three types of alloys: superalloys, stainless steel, and titanium alloys.
[66]	2022	This study presents a comprehensive material design framework based on machine learning that utilizes experimental data from LWAM Ti-6Al-4V. The framework provides multiple bead properties, such as surface quality, microstructure, bead geometry, bead dilution, and aspect ratio, simultaneously, and the models are optimized, tested with error metrics and validated through cross-validation. The proposed methodology can be used to predict the overall quality, geometry, and microstructural properties of beads across the processing space in a 3D contour plot.
[58]	2022	This research involved using AlMg4,5MnZr aluminum wire and AISI 316L stainless steel wire to develop a process for coaxial laser metal deposition welding. The results showed that linear models are useful for predicting the influence of process parameters on bead size. Finding the right combination of speed ratio and energy per unit length is important for a successful, defect-free welding process. The linear models were able to estimate the relationship between process parameters and bead width and height for both types of materials tested.
[69]	2022	The DOE method was used to perform laser cladding using three lasers and a coaxial wire-feeding system. The width of the cladding is strongly affected by laser power, while the height is affected by scanning speed. The grain size and microhardness also decrease from bottom to top. A method for automatically cutting the wire is needed to automate the process, and an overlap ratio of 31% in the height direction is appropriate for depositing thin walls.
[60]	2022	The study examined the effect of varying overlap ratios on the final product of laser wire-feed additive manufacturing of 5A06 aluminum alloy. A 3D numerical model was verified through experiments and it was determined that an overlap ratio of 20% is optimal for successful additive manufacturing, as determined by evaluating surface flatness.

**Table 3:** Summary of monitoring systems and control algorithms that are used in LWAM.

Ref.	Monitoring System	Controller Algorithm	Finding/Results
[83]	Two cameras	PI controller with feed-forward compensator	Bead height and width control in real-time can be obtained using PI controller
[84]	Camera with composite filter	PID controller	Adjusting wire feed speed for the next layer introduced resulted in process stability.
[76]	profilometer	MPC controller	A model and an MPC controller were designed to predict bead geometry in LWAM
[80]	pyrometer	closed-loop control system	The efforts of tuning the deposition process were reduced due to the designed temperature control done by the control system.
[70]	Camera and 3D scanner	Iterative learning controller	The proposed controller was able to compensate for local changes to maintain a smooth flat surface during the deposition
[71]	Light-based 3D scanner	Layer height closed loop controller	The developed controller was able to correct the local intra-layer defects and re-established the planar layers after a certain number of deposited layers.
[72]	Light-based 3D scanner	Adaptive Computer Aided Manufacturing controller	The proposed control strategy was able to stabilize the process deposition, as long as minor deviations between the last layer height and the laser focusing point were properly monitored.
[73]	Passive vision system	Self-adjusting controller for variable layer width	The results showed that the proposed controller is effective for increasing the process stability under optimal given layer width ranges between 6 to 9 mm.
[74]	Optical coherence tomography	Layer height closed loop controller	Closed-loop controller was able to track bead height through the control of the wire feed rate, to obtain a more uniform layer structure.
[75]	Near-Infrared Monochrome (NIRM) camera	MPC controller	The proposed MPC was able to keep the melt pool size at the set point under different tested process conditions with a small overshoot and a short settling time.
[77]	Thermal camera	Real-Time closed-loop melt pool size (MPS) control	Three different controller strategies were conducted. The three controllers provided a reduction in the printing time and improved the printed quality.
[80]	Thermal camera	PI controller	The controller was able to track a predefined temperature reference signal to lower the defects of the final product
[82]	CCD camera	Data-driven adaptive control strategy	The proposed controller improved geometric accuracy of the final printed metal parts with less fluctuation and better consistency.
[57]	high-speed digital camera	N/A	Best angle of wire feeding was found, and a second-order polynomial equation that related process parameters to the geometry characteristics was established.

**Table 4:** Summary of different path-planning approaches used in MAM.

Ref.	Year	Printing Setup	Approach
[85]	2016		Developed a method based on the decomposition of required geometry into sub-volumes using a curvature-based volume decomposition method. Then, the topological information is used to merge the volumes into ordered groups.
[98]	2017	6-axis robot with a 2-axis tilt and rotatory positioning system	worked on a hybrid slicing technique. This utilises the extra degrees of freedom offered by the positioning table. This approach recognizes the overhanging regions and revolves the printed object to achieve a planer printing path. This improved the accuracy of the produced structure with the CAD model.
[86]	2018	Mobile 3D printing robot	Proposed a method for generating the path for mobile printing robots. The idea was to work with more than one printing robot simultaneously without them interfering with each other. This was done by the development of a chunk-based slicing method that divides the required geometry into parts. Each one is assigned to a printing robot in order to achieve synchronized printing.
[89]	2018	Zortrax M200 3D printer	To produce a path for the printing head for printing on a non-planar surface. It was possible to fabricate lattice structures using a parent non-planer surface. In this way, there was no need to produce a support structure to fabricate the required lattice structure.
[91]	2018	6-axis robot	Developed a mixed-layer adaptive slicing method. The main idea was to use planar and non-planar slicing depending on each sub-domain of the geometry.
[99]	2018	A 6-axis robot with a 2-axis tilt and rotatory positioning system	a Matlab code was developed to generate a tool path for the robot. This path took into consideration volume slicing, contour filling, track trimming/elongation, stair-step effect compensation and post-processing.
[96]	2019	6-axis robot	Developed a unit block-based planning approach. They aimed at slicing geometries with varying layer thicknesses. The unit block is dependent on the process parameters, such as deposition rate and geometric shape. Their approach resulted in improving surface waviness, deposition rate and material utilization.
[92]	2020	6-axis robot	Introduced a spherical slicing approach. In their method the geometry is divided into a set of spherical layers that, when combined, produce the final shape. The tool path is then generated based on each layer.
[93]	2020	6-axis robot	Developed a parametric-integrated approach for path generation of the printing head.
[94]	2020	6-axis robot	Developed a layer-by-layer slicing approach, taking into account the trajectory optimization based on its limitation during printing. This approach showed improved 3D printed part accuracy with increased efficiency in the 3D printing process.
[87]	2021	Multi-axes robotics arm on moving conveyor	Method for slicing radial objects using concentric ray lines. This can be an interesting method for printing helical-shaped objects such as extruders or spiral conveyors. Their method was proposed for large-scale AM techniques to avoid using support materials during printing.
[88]	2021	Multi-axes robotics arm	Solved the slicing the problem starting with robotics kinematics. In their slicing algorithm, the tool path was generated by taking into consideration the robotics joints’ velocity limits. This method shows an improvement in the robotics workspace coverage compared with other slicing methods.
[95]	2022	A rotary 3D printer with two-DOFs translations and two-DOFs rotations	introduced a ray-based slicing approach. This method, along with helical path planning, was used to cover non-uniform path spacing between adjacent paths in the same layer.
[97]	2022	6-axis robot	Developed a slicing approach that ensures the welding takes place on a horizontal plane. This attempted to improve the bead uniformity by removing the gravity effect. In their approach, the geometry was divided into sub-regions and a path for the tool was calculated for each one.
[100]	2022	A 6-axis robot with a 2-axis tilt and rotatory positioning system	attempted to generate a tool path with consistent deposition orientation relative to gravity. Moreover, they took into consideration the ability of the path to produce an overhang structure without any need for support. They used an alternating tool path direction approach and the manipulation of the CAD model surface normal vectors.

## Abbreviations

The following abbreviations are used in this manuscript:

AAM	Arc Additive Manufacturing
CFD	Computer Fluid Dynamics
DED	Directed Energy Deposition
EBAM	Electron Beam Additive Manufacturing
FEM	Finite Element Method
LAM	Laser additive manufacturing
LBM	The Laser-Based Manufacturing
LWAM	The Laser Wire-Feed Metal Additive Manufacturing
MAM	Metal Additive Manufacturing
SLS	Selective Laser Sintering
LHW	Laser hot-wire
NAB	Nickel aluminum bronze
DEDAM	Directed energy deposition additive manufacturing
LHWD	Laser hot wire deposition
HRP-DED	High-Deposition Rate Powder-Based Laser Directed Energy Deposition
